# The Cytoplasmic LIM Domain Protein Espinas Contributes to Photoreceptor Layer Selection in the Visual System

**DOI:** 10.3390/biology9120466

**Published:** 2020-12-14

**Authors:** Alejandra Fernández-Pineda, Martí Monge-Asensio, Martín Rios, Marta Morey

**Affiliations:** 1 Departament de Genètica, Microbiologia i Estadística, Facultat de Biologia, Universitat de Barcelona, 08028 Barcelona, Spain; alexafernan@hotmail.com (A.F.-P.); martimonge95@gmail.com (M.M.-A.); mrios@ub.edu (M.R.); 2 Institut de Biomedicina (IBUB), Universitat de Barcelona, 08028 Barcelona, Spain

**Keywords:** *espinas*, *flamingo*, visual system, layer selection, R8 photoreceptor, wiring specificity

## Abstract

**Simple Summary:**

One of the central questions in neurobiology is how neurons discriminate between one another during circuit assembly. A common strategy of many nervous systems is the organization of brain regions in layers, to facilitate that neurons encounter a limited repertoire of synaptic partners. The fly visual system, which is structured in layers like many regions of the vertebrate brain, is used to identify cell surface molecules that mediate recognition between neurons and allow them to extend to specific layers. However, little is known about the intracellular pathways that link cell surface molecules to the cytoskeleton to determine whether or not to stabilize in a layer. Flamingo and its vertebrate homologs CELSR1/2 are cell surface molecules with widespread roles in neurite growth. In the fly visual system in particular, Flamingo regulates layer selection of a particular neuronal type. Our data suggests that in this context, Flamingo signals to the cytoskeleton through the conserved cytoplasmic molecule Espinas/PRICKLE2. Given that Flamingo and Espinas, as well as their respective vertebrate homologs, are broadly expressed in the nervous system, elucidating the interactions between them can reveal conserved mechanisms and provide valuable insights into the assembly of neural circuits.

**Abstract:**

During circuit assembly it is essential that neurons connect with their specific synaptic partners. To facilitate this process, a common strategy in many organisms is the organization of brain regions, including the fly visual system, in layers and columns. The atypical-cadherin Flamingo (Fmi) and the receptor Golden Goal (Gogo) were proposed to regulate both the temporary and final layer selection of the R8 photoreceptor, through the cytoplasmic domain of Gogo. Our data suggests that Fmi intracellular signaling is also relevant for R8 final layer selection. The LIM-domain cytoplasmic molecule Espinas (Esn) binds Fmi, and they cooperatively control dendritic self-avoidance in sensory neurons. We observed defects in R8 layer selection in *esn* mutants with axons overshooting the final target layer, and we demonstrated that the LIM domain is necessary for layer selection. *fmi* knockdown in photoreceptors results in most R8 axons stalling at the temporary layer, however, we also detected R8 axons projecting past the final-target layer, and showed that *fmi* and *esn* genetically interact. Based on the previously described physical and genetic interactions between Fmi/Esn and the findings presented here, we propose that Esn signals downstream of Fmi to stabilize R8 axons in their final target layer.

## 1. Introduction

One of the central questions in neurobiology is how neurons discriminate between one another during circuit assembly, to achieve the exquisite synaptic specificity that makes each circuit functionally unique. A common strategy of many nervous systems is the organization of brain regions in layers and columns [1,2,3,4]. The formation of these structures takes place during development in a sequential manner as new neurons are added to the system, which restricts and facilitates the possible interactions between neuronal processes at any given time. In some cases, specificity can be achieved by developmental rules [5,6,7,8], such as for example coordinated growth of synaptic partners. However, in many instances intercellular recognition interactions play an important role. Different types of intercellular interactions and molecular mechanisms can result in synaptic specificity [9]. For example, selective recognition between synaptic partners that express ligand/receptor proteins can facilitate adhesion and the formation of synapses between neurons. Alternatively, among other mechanisms, specificity can be also achieved by cell–cell interactions resulting in repulsion, and hence, a decreased presence of synapses between these two neurons. Much effort has been put into identifying cell surface molecules that mediate cell recognition, and elucidating their contribution to specificity [9]. However, much less is known about the specific signaling pathways downstream and how they modulate axonal behavior.

**Figure 1:**
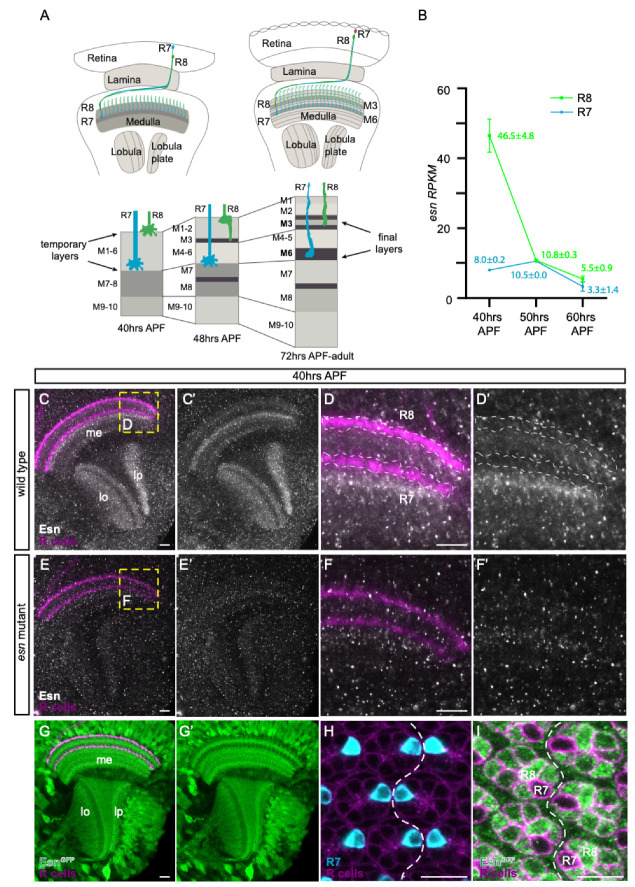
Espinas (Esn) expression in the optic lobe and photoreceptors. (**A**) Schematic showing optic lobes and the photoreceptor array from 40 h after pupal formation (APF) onwards to the adult stage. On the left, the optic lobe at 40 h APF, when all R8 axons (green) sit in their temporary layer at the edge of the medulla. On the right, the optic lobe in the adult stage when all R8 axons have extended to their final target layer, M3. Below, a detail of the medulla showing R8 and R7 layer selection in parallel to layer formation, which at 72 h APF reaches what will be the adult structure. (**B**) Graph showing expression in Reads Per Kilobase Million (RPKM) of *esn* in the R8 (green) and R7 (cyan) neurons in three different pupal stages. (**C**–**I**) All images are confocal sections taken from 40 h APF brains. Photoreceptors (R cells) are labeled with anti-Chaopin antibody except for panel H, where the genetic marker *GMR-myr-tdtom* is used. (**C**–**F’**) Esn expression (gray) using anti-Esn antibody in wild type (**C**–**D’**) and *esn* mutant (**E**–**F’**) optic lobes. Yellow dashed boxes in (**C**,**E**), respectively, show the magnified area in (**D**,**F**). Dashed regions of interest in (**D**,**D’**) show the position of R8 and R7 axons. (**G**,**G’**) Esn expression (green) in the optic lobe using the protein trap line *esn^MI03075-GFSTF.2^*. (**H**) Retinal section showing photoreceptor cell bodies (magenta) and the equator (white dashed line). This is the dorso-ventral boundary where the mirror image organization of the photoreceptors in the ommatidia is evident. R7 photoreceptor cell bodies (cyan) face each other across the equator. (**I**) Esn expression (green) observed with the protein trap in photoreceptor cell bodies (magenta). The R7 and R8 photoreceptor cell bodies are indicated. me, medulla; lo, lobula; lp, lobula plate. Scale bars 10 microns.

The fly eye is composed of some 750 units or ommatidia. Each ommatidium contains eight photoreceptor cells: R1–6 are involved in motion detection and terminate in the lamina, the first neuropil of the visual system; R7 and R8 are involved in color vision and extend to the next neuropil, the medulla (Figure 1A). Similar to vertebrates, the fly visual system is structured in columns and layers [2,3]. The spatial representation of the visual input provided by photoreceptors is morphologically supported by parallel columnar synaptic modules that process the information from discrete adjacent points of the visual field, in the lamina and medulla neuropils. Orthogonal to this columnar organization, the medulla is divided in 10 layers (M1–10). The R8 terminates in M3, and the R7 in M6 (Figure 1A). The fact that these two functionally and developmentally related neurons have unique layer selection makes them excellent candidates to identify genes contributing to their differential layer selection [10,11].

The R8 photoreceptor is the first one to project into the brain, and it does so in two steps [12]. In larval stages, R8 axons from the different ommatidia must retain their positional information in the eye. For that, they need to distribute evenly along the anteroposterior and dorsoventral axes to form a retinotopic map when they exit the optic stalk, the nerve that connects the eye disc with the brain. In this first step, R8 axons enter through the prospective lamina targeting their specific synaptic columnar units, and continuing to reach the edge the medulla where they sit in the temporary layer, that will be called M1 in the adult. In a second step, in midpupal stages, R8 axons need to detach from M1 and extend filopodia towards the M3 final target layer, initiating the process that will transform the growth cone into a synaptic terminal [13] (Figure 1A).

Several studies have identified various cell surface molecules involved in the establishment of retinotopy and R8 layer selection. The atypical-cadherin Flamingo (Fmi) and the transmembrane protein Golden Goal (Gogo) mediate axon–axon interactions between R8 processes as they exit the optic stalk in a bundle. These proteins facilitate the defasciculation of R8 cells from the bundle so that they enter the lamina evenly spaced maintaining their retinotopy [14,15,16]. These two molecules also cooperate in R8 layer selection [15,16,17]. It was initially proposed that Fmi and Gogo regulated both the R8 temporary and final layer selection, and that the intracellular signaling for layer selection was mainly mediated by the cytoplasmic domain of Gogo while Fmi signaling was dispensable [17]. A recent study by the same group [18] refines this model showing that Gogo and Fmi cooperate to guide the R8 into the right in place in the column; Gogo counteracts Fmi to inhibit the formation of filopodia; that in midpupal development when R8 axons extend filopodia to their final target layer they only express Fmi. The latter finding warrants revisiting the initial idea that Fmi intracellular signaling is not required for R8 final layer selection.

Supporting the idea that Fmi intracellular signaling is relevant for R8 final layer selection, we show that the cytoplasmic LIM domain protein Espinas (Esn) is also involved in this process. Esn is one of the members of the Drosophila PET-LIM domain family, composed by Prickle (Pk), Espinas, and Testin [19]. It has been demonstrated that the LIM domain of Esn binds to the juxtamembrane domain of Fmi, and that both proteins cooperatively control dendritic self-avoidance in sensory Da neurons [20]. Here, we find that the cytoplasmic LIM domain protein Esn is enriched in R8 compared to the R7 photoreceptors in pupal stages. We identify defects in R8 final layer selection with axons overshooting their final target layer in *esn* mutants, and we demonstrate that the LIM domain is essential for Esn layer selection function. *fmi* knockdown in photoreceptors results in most R8 axons stalling at the M1 layer, however, we also detect R8 axons projecting past the M3 layer, and a genetic interaction between *fmi* and *esn*. Based on the previously described physical and genetic interactions between Fmi/Esn [20] and our findings, we propose that Esn signals downstream of Fmi in the R8 cell to stabilize the R8 axon in the M3 layer.

## 2. Materials and Methods

### 2.1. Genetics

Flies were grown in standard medium at 25 °C except for RNAi experiments and related controls, which were performed at 29 °C. All genotypes analyzed are specified in detail in the Appendix A. Stocks used in this study were *esn^MI03075-GFSTF.2^* (BDSC65325, [21,22]); *w*; *GMR-myr-tdtom, prosDSCP-p65AD*; *44F08-gal4DBD, UAS-RpL10* (R7 marker [11]); *Rh6-lacZ* (BDSC8120); *esn^f00447^* (BDSC18331, [23]); *esn^KO6^* (a gift from Tadashi Uemura, [20]); *Df(2R)BSC262* (BDSC23297); *Df(2R)Exel6283* (BDSC7748); *GMR-gal4, UAS-Dcr2, Rh6-lacZ* and *GMR-gal4, Rh6-lacZ* recombinant chromosomes (a gift from S.Lawrence Zipursky); *UAS-esnRNAi* (transformant ID: 30037/construct ID: 7841 VDRC); *UAS-esn* and *UAS-esnΔLIM* (this study); *sens-gal4, UAS-UtrnCHGFP* (a gift from Orkun Akin, [13]); *UAS-fmiRNAi* (a gift from Iris Salecker, [24]); *fmi^E59^* (BDSC41776, [25]).

### 2.2. DNA Constructs

*pUAST-6xFlag* plasmids containing isoform A of *esn* with and without the LIM domain (an internal deletion of amino acid residues between 243 and 423) were obtained from the original source [20]. To generate site directed integration transgenics flies for rescue experiments we used EcoRI and KpnI enzymes to obtain *6xFlag esn* and *6xFlag esnΔLIM* fragments that were cloned into the *pUASTattB* vector [26] using the same restriction enzyme sites. The final constructs were injected into the *attp2* (25C6) landing site on the 3rd chromosome.

### 2.3. Immunohistochemistry

Fly brains were dissected in PBS and fixed in 4% PFA in PBL (75 mM lysine, 37 mM sodium phosphate buffer, pH 7.4) for 25 min. After fixation, the tissue was washed in PBS with 0.5%Triton-X-100 (PBST) and blocked with PBST with 10% normal goat serum. Primary and secondary antibody incubations were performed in PBST and 10% normal goat serum, typically overnight at 4 °C. The following primary antibodies were used for immunohistochemistry: guinea pig anti-Esn (1:1000, [20]), mouse anti-Chaoptin (1:50, 24B10, Developmental Studies Hybridoma Bank, DSHB), chicken anti-GFP (1:800, ab13970, Abcam, Cambridge, UK), rabbit anti-β-galactosidase (1:1000, 0855976, Cappel, Malvern, PA, USA). Appropriate Alexa Fluor 488 and 568 secondary antibodies (Life Technologies, Carlsbad, CA, USA) were used at 1:500 concentration. Brains were mounted for confocal microscopy in Vectashield (Vector Laboratories, Burlingame, CA, USA). Samples were analyzed with a Leica TCS SP2 and SPE confocal microscopes. Fiji [27] was used to process confocal images and figures were assembled using Adobe Illustrator (Adobe, San Jose, CA, USA).

### 2.4. Photoreceptor Phenotype Quantification and Statistics

For each brain analyzed, we quantified 60–80 μm of tissue in each optic lobe. The total number of Rh6 expressing R8 axons was counted, and among them the ones with R8 layer selection defects. For each brain a percentage of mistargeted axons was quantified. The number of brains, and total number of R8 axons quantified per genotype was always above six brains and over 800 total R8 axons. Exact numbers for each genotype are indicated in the Appendix A. The mean and standard deviation (SD) of the percentage of mistargeted R8 axons were obtained. We performed one-way ANOVA followed by Tukey’s post hoc test to obtain *p*-values. *p*-values for pairwise comparisons relevant to our biological inquiry are shown in the bar graphs. Statistical analysis was carried out using Prism 6 (GraphPad Software Inc., San Diego, CA, USA).

## 3. Results

### 3.1. esn Is Expressed in the Optic Lobe and Enriched in R8 versus R7 Photoreceptors

In previously generated developmental transcriptomes of the R8 and R7 photoreceptors [11], we observed that *esn* was enriched in R8 compared to R7 cells at 40 h after pupal formation (APF) (Figure 1B). This is some hours before R8 cells proceed to their final target layer, which will be called M3 in the adult [12,13]. We first used anti-Esn antibody to analyze expression in R8 and R7 photoreceptor growth cones in the medulla of 40 h APF animals since these structures sense the environment and Fmi is expressed in them [14,15,18]. We observed an Esn layered pattern in the medulla and lobula, as well as signal in the lobula plate (Figure 1C,C’). However, we could not detect any signal difference between R8 and R7 growth cones (Figure 1D,D’), suggesting that Esn protein levels in the R8 were probably below the antibody detection limit. It actually looked like there was a dip in the signal where R8 and R7 growth cones were, while there was a faint signal in between the R8 and R7 growth cones, and stronger signal below R7 growth cones (Figure 1D’). These patterns were strongly reduced in the mutant background (for details on the mutant see next section) (Figure 1E–F’). We analyzed Esn expression at later stages, between 48 and 70 h APF, when the R8 extends a filopodia to its final target layer and transitions into a synaptic terminal (Figure S1A–C’). Due to the expression of Esn in neurites between the R8 and the R7 final target layers we could not discern expression in the R8 growth cone/axon from expression in other neurons.

We next tried a Minos-mediated integration cassette (MiMIC)-based Esn protein trap line [21,22]. In these lines, the original gene trap cassette [28] has been replaced by a protein trap cassette, which creates a new exon that includes the eGFP coding sequence in frame with the rest of the protein. Based on the Minos transposon insertion site, the protein trap cassette should introduce the eGFP 327 amino acid (aa) residues downstream of the ATG of longest isoform (esn-PC, 1134aa), and position the eGFP upstream of the ATG of the other three smaller isoforms (esn-PA, -PB, 785aa; esn-PD, 851aa). With this protein trap line, Esn appeared to be widely expressed in brain at 40 h APF. In the optic lobe we could observe expression in many cell bodies, a layered pattern in the medulla and lobula reminiscent of the one observed with the antibody, as well as expression in the lobula plate (Figure 1G). The lower expression of Esn where R8 and R7 axons terminate and high expression between their axons was clearly observed with this line (Figure 1G’). We observed expression in cell bodies in the medulla which we had not detected with the antibody. Since the extensive medulla signal made it difficult to evaluate expression in R8 versus R7 growth cones, we analyzed expression in the retina where the different photoreceptor cell bodies lie and can be easily identified (Figure 1H,I). We observed different levels of Esn expression in R1-R6 photoreceptors. While the R8 had high levels of Esn expression, the expression in the R7 was clearly the lowest. Analysis of retinas at 48–50 h APF, when the R8 axon extends the filopodia, shows that Esn is indeed expressed in the R8 during this process (Figure S1D–E’).

Thus, consistent with the transcriptome expression data, we observed a striking differential expression of Esn between the R8 and the R7 cell.

### 3.2. R8 Photoreceptor Axons in esn Mutant Animals Mistarget Past Their Final Target Layer

**Figure 2:**
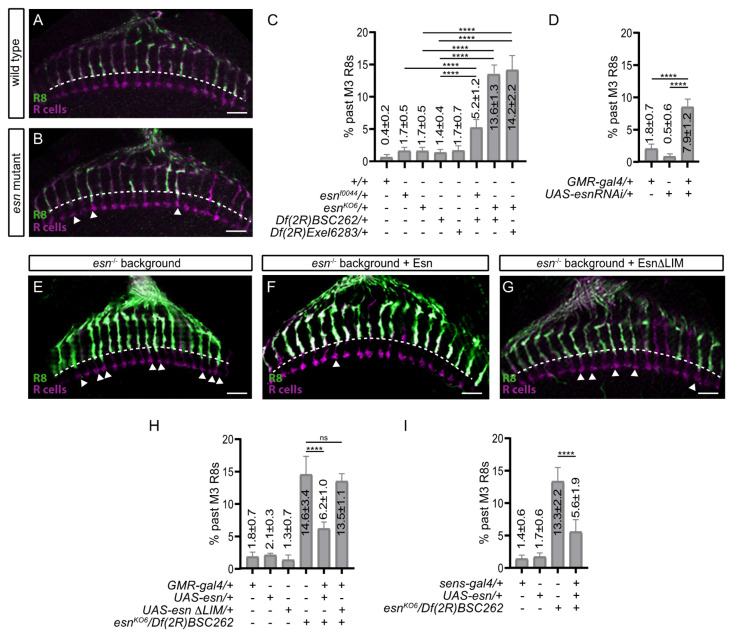
*esn* requirement in R8 final layer selection. All images are confocal sections of adult optic lobes with all photoreceptor axons (magenta) labeled with anti-Chaoptin and R8 axons (green) labeled with anti-lacZ antibody against the R8 specific marker *Rh6-lacZ*. Arrowheads indicate columns where R8 axons extend past their final target layer, M3 (white dashed line). Graphs show quantification of phenotypes and the statistical significance of relevant comparisons. (**A**) In control animals R8 axons terminate in the M3 layer. (**B**) In *esn* mutant animals, R8 axons in several columns extend past the M3 layer. (**C**) Graph showing the percentage of mistargeted R8 axons that extend past the M3 layer for different control and mutant allelic combinations. (**D**) Graph showing the percentage of mistargeted R8 axons in all-photoreceptor (*GMR-gal4*) specific RNAi *esn* knockdown. (**E**–**G**) Confocal sections of adult arrays in an all-photoreceptor specific rescue experiment: (**E**) mutant background, (**F**) rescue experiment with a full length *esn* construct, and (**G**) rescue experiment with an *esn* construct lacking the LIM domain. (**H**) Graph showing the quantification of mistargeted R8 axons in the all-photoreceptor specific rescue experiment. (**I**) Graph showing the quantification of an R8-specific (*sens-gal4*) rescue experiment. **** *p* < 0.0001. Scale bars 10 microns.

**Figure 3:**
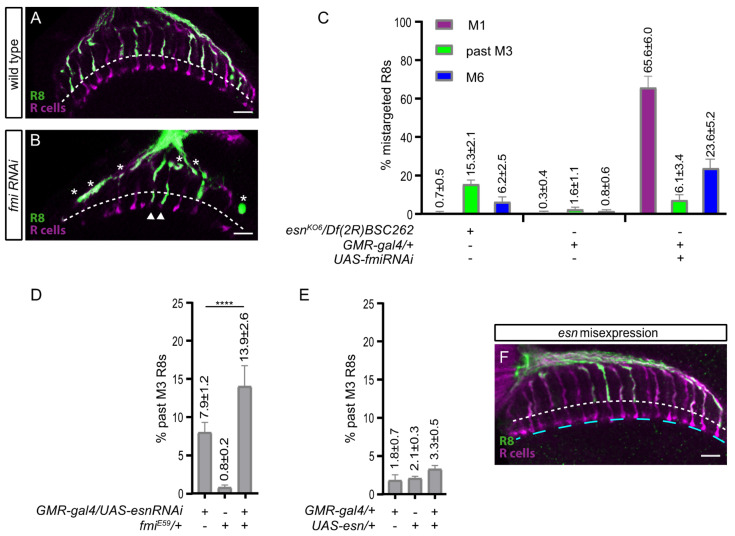
Contribution of the *esn/fmi* genetic interaction to R8 final layer selection. All images are confocal sections of adult photoreceptor arrays (magenta) with labeled R8 axons (green) as in Figure 2. (**A**) Control animals with R8 axons terminating in the M3 layer (dashed line). (**B**) Array of an all-photoreceptor *fmi* knockdown animal. Asterisks mark R8 axons that get stuck in the M1 layer at the edge of the medulla. Arrowheads mark two columns where R8 axons extend past the M3 layer, almost to the M6 layer, where R7 axons (magenta) terminate. (**C**) Graph showing the percentages of mistargeted R8 axons to the M1 layer, past the M3 layer, and almost to the M6 layer in the *esn* mutant; a control for the *fmi* RNAi knockdown, and the *fmi* RNAi knockdown. (**D**) Graph showing the increase in the percentage of past M3 mistargeted R8 axons observed as a result of the *esn/fmi* genetic interaction. (**E**) Graph showing that overexpression of *esn* in the R8 does not affect R8 layer selection. The small percentage of mistargeted R8 axons observed is a result from the additive effect of heterozygous controls. (**F**) Adult array of an animal expressing *esn* in all-photoreceptors. R8 axons terminate in the M3 layer (white dashed line). R7 axons terminate in the M6 layer (lower cyan dashed line). **** *p* < 0.0001. Scale bars 10 microns.

To investigate the role of *esn* in R8 layer selection we analyzed the adult photoreceptor array of control and mutant allelic combinations. To visualize the R8 axons we used a cell type specific opsin reporter (*Rh6-lacZ*), which labels 70% of R8 photoreceptors. In wild type animals and heterozygous controls, the great majority of R8 axons terminate in the M3 layer (Figure 2A,C). In *esn* mutants, which are viable, a percentage of R8 axons overshot the M3 layer, that is they extended past it, terminating half way between the M3 and M6 final target layers (Figure 2B,C). This phenotype was fully penetrant, and for the hypomorphic insertion allele *esn^f00447^* against *Df(2R)BSC262* mistargeted R8 axons were 5.2% ± 1.2 SD, while for the knockout allele *esn^KO6^* against the same deficiency mistargeted R8 axons were 13.6% ± 1.3 SD. The knockout allele against another deficiency (*Df(2R)Exe*l6283) resulted in a very similar percentage (14.2% ± 2.2 SD). It is worth mentioning that there were a few examples of R8 mistargeted axons extending almost to the M6 layer, where the R7 axon terminates, in both knockout allele combinations (see later in the text and in Figure 3C). The *esn* mutant background that we used in the rest of our experiments was *esn^KO6^/Df(2R)BSC262*.

In summary, while the percentage of mistargeted R8 axons varied depending on the allelic combination, R8 axons overshooting the M3 layer were consistently observed in *esn* mutants.

### 3.3. esn Is Required in the R8 Photoreceptor for Final Layer Selection

Given the wide *esn* expression in the optic lobe, we next tested whether we could recapitulate the R8 mistargeting phenotype knocking down *esn* exclusively in photoreceptors. To this end we used the *GMR-gal4* driver, which is expressed in all photoreceptors, to express a *UAS-esnRNAi* line and we indeed observed 7.9% ± 1.2 SD of R8 axons overshooting the M3 layer (Figure 2D). Considering the percentage of mistargeted R8 axons contributed by the *GMR-gal4* (1.8%) and *UAS-esnRNAi* (0.5%) chromosomes, the knock down phenotype (7.9% − (1.8% + 0.5%) = 5.6%) is very similar to the mistargeting percentage obtained in the *esn^f0044^*/*Df(2R)BSC*262 hypomorphic allelic combination (5.2%). While this experiment suggested that *esn* was required in photoreceptors for R8 final layer selection, it did not distinguish whether the low percentage of mistargeted R8 axons was due to a low efficiency of the RNAi or the possible contribution of *esn* expressing medulla neurons to the R8 mistargeting phenotype. To address the latter, we performed a photoreceptor specific rescue experiment in the *esn* mutant background (Figure 2E,F,H). Using *GMR-gal4* to express *UAS-esn* in the mutant background resulted in 6.2% ± 1.0 SD of mistargeted R8 axons, and thus, a rescue compared to 14.6% ± 3.4 SD of mistargeted R8s found in the mutant. The *GMR-gal4* and *UAS-esn* transgenes result in a small but consistent percentage of mistargeted R8 axons: 1.8% ± 0.7 SD and 2.1% ± 0.3 SD, respectively. Subtracting this contribution from the rescue value (6.2% − (1.8% from *GMR-gal4* + 2.1% from *UAS-esn*)) results in 2.3% of mistargeted R8s in the rescue experiment. Hence, one can calculate the rescue percentage to be, indeed, of around 84% (14.6% mistargeted R8s in the mutant vs. 2.3% mistargeted R8s in the *GMR* rescue). We also performed a rescue experiment using the *sens-gal4* driver, which is expressed in the R8 cell and has a low residual expression in the R2 and R5 photoreceptors (Figure 2I). The rescue percentage observed was of similar magnitude: 81% (13.3% mistargeted R8s in the mutant vs. 2.5% of mistargeted R8s in the *sens* rescue (5.6% − (1.4% from *sens-gal4* +1.7% from *UAS-sens*)).

Thus, the photoreceptor specific knockdown recapitulation of the *esn* mutant R8 mistargeting phenotype, together with the strong R8 specific rescue in the mutant background show that *esn* is both necessary and sufficient for R8 final layer selection. In addition, the similarity between the all photoreceptor (*GMR-gal4*) and R8 photoreceptor (*sens-gal4*) rescues indicates that it is highly likely that *esn* is autonomously required in the R8 for final layer selection, with little if any contribution from the R1–6 photoreceptors that express Esn. Although these are strong photoreceptor specific rescues, we cannot fully discard the possibility that a small part of the R8 mistargeting phenotype is nonautonomous and caused by the requirement of *esn* in medulla neurons.

### 3.4. An esn/fmi Interaction Contributes R8 Final Layer Selection but esn Is Not Instructive

The Esn LIM domain has been shown to physically interact with the intracellular juxtamembrane domain of Fmi [20], thus, we wondered whether this domain mediated *esn* function in the R8 cell. To address this question, we used *GMR-gal4* to express Esn protein lacking the LIM domain (*UAS-esnΔLIM*) in the *esn* mutant background (Figure 2G,H). We observed no difference between the percentage of mistargeted R8s in the mutant (13.6% ± 1.3 SD) and the rescue experiment (13.5 ± 1.1 SD), which indicates that the Esn LIM domain is essential for the role of Esn in R8 final layer selection.

Given that *fmi* regulates R8 layer selection [15,17], and that *fmi* and *esn* interplay has been shown essential in other neurons [20], we wondered if *fmi* and *esn* also cooperated in R8 final layer selection. In mosaic animals it has been reported that a big percentage of the single *fmi* mutant R8 axons stop at the M1 temporary layer [17]. Alternatively, in *esn* mutant animals most R8 axons extending past the M3 layer terminate in between the R8 and R7 layers (Figure 2B,C). In order to reconcile these two different phenotypes, we used the *GMR-gal4* driver to knock down *fmi* and performed a detailed phenotype analysis of R8 axons (Figure 3A–C). We observed that, similar to mosaic experiments [17], 65.6% ± 6.0 SD of R8 axons terminated in their temporary layer at the edge of the medulla. Interestingly, we also observed that 6.1% ± 3.4 SD of R8 axons overshot the M3 layer terminating between the R8 and R7 and that 23.6% ± 5.2 SD were almost at the M6 layer. Indeed, in parallel analysis of the mutant showed that besides the R8 axons terminating between the M3 and M6 layers (15.3% ± 2.1 SD), there were 6.2% ± 2.5 SD R8 axons that almost reached the M6 layer (Figure 3C). A possible redundancy with the paralog gene *pk*, which genetically interacts with *fmi* in the context of planar cell polarity [29,30], could explain why few R8 axons almost reach M6 in *esn* mutants, while many more do so in *fmi* mutants. It is of note that in our analysis of *pk* mutant alleles we also observed R8 axons overshooting past the M3 layer (Figure S2). In summary, both *fmi* and *esn* mutations show R8 axons failing to stabilize in the M3 layer and extending past it, or almost reaching the M6 layer.

To address a possible functional cooperation between Fmi/Esn in M3 final layer selection we assessed if *esn* loss of function phenotype could be modified by reducing *fmi* dosage with the nonsense null allele *fmi^E59^* (Figure 3D). This genetic background allowed for the analysis of M3 layer selection without affecting Fmi function in the M1 layer. We used the *esn* RNAi knockdown phenotype as a starting point (7.9% ± 1.2 SD), and observed that removing one copy of *fmi* had a synergistic effect, resulting in almost double the mistargeted R8s (13.9 ± 2.6 SD).

Given that Fmi is expressed both in the R7 and R8 photoreceptors, the striking difference between R7 and R8 *esn* expression pattern led us to test the possibility that *esn* misexpression in the R7 resulted in axons terminating in the M3 rather than M6 layer. Using *GMR-gal4* to express *esn* we did not detect any defects in R7 or R8 layer selection (Figure 3E,F). Thus, while *esn* is necessary, it is not instructive to mediate M3 final layer selection.

In summary, based on these experiments, we propose that Fmi and Esn could functionally interact through the Esn LIM domain to stabilize the R8 axon at the M3 layer.

## 4. Discussion

R8 and R7 photoreceptors are an excellent paradigm to address the molecular mechanisms behind differential layer selection. Transcriptional control plays a critical role in this process. The transcription factor Sequoia regulates the initial segregation of R7 and R8 growth cones in the first targeting step through relative temporal differences in its expression [7]. In the second step, unique combinations of transcription factors, Senseless and Orthodenticle in the R8 and NF-YC and Prospero in the R7, mediate final layer selection [10,31]. Given the existence of these cell type specific genetic programs, it is reasonable to hypothesize that differentially expressed genes could have a significant contribution to differential layer selection. Especially because although Fmi and Gogo are both expressed in the R8 and R7 axons they are only required for R8 layer selection [14,15,16]. Thus, finding that Esn, a LIM domain protein shown to physically and genetically interact with Fmi [20], was significantly enriched in the R8 versus the R7 cell prior the start of the second step of targeting [11], suggested two things: (1) that signaling through Fmi/Esn could be required for R8 final layer selection, and (2) that this R8 specific intracellular signaling downstream of Fmi could be instructive in the differential layer selection of the R7 and R8 photoreceptors.

**Figure 4:**
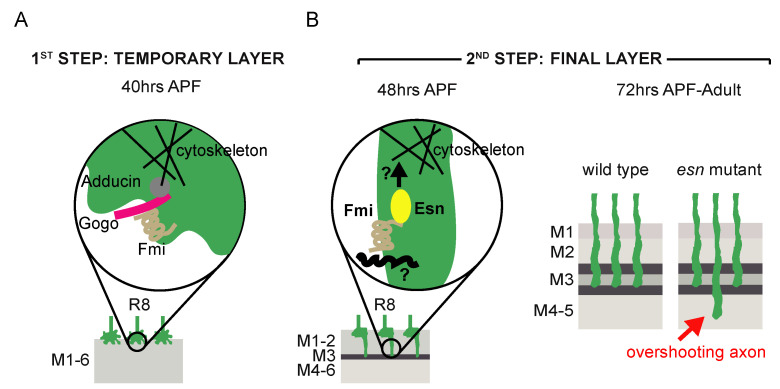
A model for R8 layer selection. (**A**) Molecular machinery present in the first step of R8 layer selection when growth cones reach their temporary layer. Gogo and Fmi interact in *cis*. The cytoplasmic domain of Gogo mediates signaling that modulates the cytoskeleton and Fmi function [17,18,32]. (**B**) Model proposed for the second step of R8 layer selection when R8 growth cones extend to their final target layer and transform into synaptic terminals. We propose that Fmi could signal through Esn to modulate the cytoskeleton and mediate stabilization in the final target layer. Most probably other cell surface molecules, acting in *cis* with Fmi and/or in parallel, are involved in this process.

Our data shows that *esn* is involved in R8 final layer selection. We showed that Esn is expressed in the R8 at the transcriptional and protein level at 40 h APF (Figure 1B,I) when it sits in the temporary layer, and at the protein level at 48–50 h APF when the R8 extends a filopodia towards the final layer (Figure S1D,E’). Our analysis with the protein trap does not clarify the subcellular localization of Esn in the R8. One possibility is that this reporter faithfully recapitulates Esn subcellular localization in the R8 and medulla cell bodies, and that expression in the R8 growth cone is hard to detect. Although using the antibody we did not see expression of Esn in cell bodies in the optic lobe, others have detected the protein in embryonic sensory organ precursors and sensory neuron cell bodies with the antibody [20]. Alternatively, it has been reported that sometimes the splicing machinery skips the donor site in the protein trap cassette generating an aberrant splicing and a mislocalized truncated protein. Still, these lines are still useful to identify in which cell types the protein is expressed. Regardless of which of these two possibilities applies to the protein trap line, its expression pattern is consistent with the transcriptomic data. We identified R8 final layer selection defects in *esn* mutants and showed that *esn* is required in the R8 photoreceptor. In addition, rescue experiments indicate that the Fmi-interacting Esn LIM domain is necessary for R8 final layer selection. While the main R8 axon phenotype in *fmi* knockdowns or mutant R8s is stalling at the M1 temporary layer, we observed axons extending past the final M3 layer, which uncovers a role for *fmi* in R8 final layer selection. Besides sharing the past M3 phenotype, we observed that *fmi* and *esn* genetically interact. These findings, together with previously described physical and genetic interactions between Fmi/Esn in Da sensory neurons [20], leads us to propose that signaling through Fmi/Esn is required for R8 final layer selection (Figure 4).

A previous study had suggested that Fmi and Gogo worked together to regulate both temporary and final layer selection [17]. There, structure–function rescue experiments suggested that in the Fmi/Gogo interaction, the intracellular signaling for layer selection was mainly mediated by the cytoplasmic domain of Gogo. On the one hand, the *gogo* mutant phenotype could not be rescued by a Gogo protein lacking the cytoplasmic domain, while the *fmi* mutant phenotype was rescued with a Fmi protein that lacked its intracellular domain [17]. We believe that the discrepancy between these previously published data and the Fmi/Esn signaling we propose has the following explanation: in the Fmi construct [33] used to test the requirement of its intracellular domain in R8 layer selection [17] there is still part of the juxtamembrane (JM) domain that binds Esn [20]. The C-terminal cytoplasmic tail of Fmi contains a highly conserved JM domain that was used as a bait in the yeast two-hybrid screen that identified Esn [20]. Further dissection analyses of the JM domain determined that the N-terminal subdomain (JM-A) bound to LIM much more strongly than the C-terminal JM-B subdomain [20]. The *FmiΔintra* transgene [33] used in the R8 study [17] eliminates the entire intracellular domain except for the first 30 amino acids, which are retained. Interestingly, these are the first 30 out of the 100 amino acids of the JM-A, and the most highly conserved [20]. Hence, we hypothesize that this remaining stretch of amino acids in *FmiΔintra* transgene [33] could allow for Esn binding, and for that reason, contribute to the reported rescue of the *fmi* mutant R8 mistargeting phenotype [17]. Moreover, the recent finding that Gogo is indeed not detected at the time of axon extension to the M3 layer but Fmi is [18], is consistent with the possibility that Fmi signals through Esn during this final layer selection period as we propose.

Fmi and its vertebrate homologs, the CELSR proteins, play central roles in both planar cell polarity (PCP) and the regulation of neurite growth. While molecular interactions between Fmi and other members of the PCP signaling pathway have been characterized in detail in planar polarity, much less is known about the molecules that interact with Fmi *in cis* within the neuronal plasma membrane or signal downstream in neurons. There is evidence of other members of the PCP pathway regulating axonal targeting and branching in the mushroom body [34,35], axonal growth [36], and dendritic self-avoidance in sensory neurons [11]. Among them is *pk*, which is the closest paralog to *esn*. Interestingly, *pk* has been shown to modulate microtubule polarity and axonal transport [37], providing a molecular link to the cytoskeleton. So far, no R8 layer selection phenotypes have been reported for members of the PCP pathway [17]. Fmi interacts *in cis* with Gogo in the first step of layer selection [17], and a direct physical interaction between Gogo and the *Drosophila* homolog of Adducin links the cell surface molecule to the cytoskeleton [32] (Figure 4A). For the second step of R8 layer selection targeting, Gogo is not expressed [18], and thus it does not interact with Fmi, although other cell surface molecules might (see below). With regards to signaling downstream of Fmi, we speculate that the role of Pk in axonal growth could be redundant to Esn function, and explain why the *esn* phenotype is limited to a percentage of mistargeted R8 axons ranging from some 13% (past M3, Figure 2C,H,I) to 21% (past M3 + M6, Figure 3C) rather than higher. The identification of Esn opens up the door to exploring signaling downstream of Fmi in the R8 photoreceptor (Figure 4B) and revisiting the possible role of *pk* in R8 layer selection (Figure S2).

Contrary to our initial hypothesis, the differential R8 versus R7 layer selection does not seem to be mediated by the differential expression of Esn in these cells. If that was the case, misexpression of *esn* in R7 cells would have resulted in their targeting to the M3 layer as R8 cells do. We did not detect any defects in R7 layer selection. Many nonmutually exclusive possibilities arise for this result. One is that Fmi in the R7 cell might simply not be competent to signal through Esn. Another is that Esn might not be able to bind to Fmi in the R7 cell. Alternatively, and maybe more likely, R8 might use a unique combinatorial code of cell surface molecules and their respective downstream signaling to extend to and stabilize in the M3 final target layer (Figure 4B). Indeed, other molecules expressed in the R8 but not the R7 have been involved in the second step of R8 layer selection. For example, the L3 lamina neuron, which at this pupal stage is in the prospective M3 layer, secretes Netrin which is recognized by Frazzled receptor in the R8 axons [13,38,39]. In this context this interaction is not related to chemoattraction but rather mediates adhesion to neuronal processes or the extracellular matrix in the target layer [13]. Several roles for the R8 specific Leucine Rich Repeat cell surface molecule Capricious have been proposed: consolidation of R8 growth cones in the temporary layer upon entering the medulla [40]; final layer recognition through homophilic adhesive afferent–target interactions, since it is also expressed in the medulla [41]; or interactions with a yet unidentified heterophilic ligand [42]. Redundancy in the molecular mechanisms regulating R8 final target layer selection, can also explain the limited percentage of mistargeted R8 axons in *esn* mutants.

*fmi* and *esn* are broadly expressed in the fly nervous system, and their respective vertebrate homologs, CELSR1/2 and PRICKLE2, as well [43,44]. Thus, elucidating the interactions between them can reveal conserved mechanisms and provide valuable insights into the development of the nervous system.

In addition to elucidating how molecules interact to achieve wiring specificity, one of the next challenges in the field is to understand how wiring defects impact the functionality of circuits. While with light microscopy we can address whether there is an increase or decrease in synapse number, and in some cases, we can assess whether postsynaptic partners are the expected ones, the biological meaning behind the observed change is hard to infer. For example, we do not know if the reduction or the increase in synaptic number observed is enough to cause a functional change, if new presynaptic sites are orphan or are paired with postsynaptic sites in other neurons, if these other neurons are cognate synaptic partners or not, etc. The community is heavily invested in making progress on several of these fronts. Massive efforts are being carried out to reconstruct neural circuits at the EM level in the fly visual system [45]. Complementing this data with chemoconnectomes [46] will facilitate functional modeling and will open the door to predictions that could be later functionally tested, either through electrophysiology or behavior. When these tools are available it will be the perfect time to revisit wiring mutants and assess the functional consequence of their wiring defects.

## Supplementary Materials

The following are available online at https://www.mdpi.com/2079-7737/9/12/466/s1, Figure S1: Extended Espinas (Esn) expression analysis, Figure S2: Analysis of *prickle* (*pk*) mutant alleles.

## Appendix A

Genotypes and quantification data.

Figure 1

-1C, C’, D, D’: *w*; +; +

-1E, E’, F, F’: *w*; *esnKO6/Df(2R)BSC262*; +

-1G, G’, I: *yw*; *esnMI03075-GFSTF.2/+*; +

-1H: *w*; *GMR-myr-tdtom*, *pros-DSCP-p65AD/*+; *44F08-gal4DBD*, *UAS-RpL10/*+

Figure 2

-2A: *w*; +; *Rh6-lacZ/*+

-2B: *w*; *esnKO6/Df(2R)BSC262*; *Rh6-lacZ/*+

-2C: graph bars from left to right; N = brains, R8 = total R8s counted in N brains

*w*; +; *Rh6-lacZ/+*            N = 7; R8 = 1211

*w*; *esn^f0044^/+*; *Rh6-lacZ/+*            N = 8; R8 = 1236

*w*; *esn^KO6^/+*; *Rh6-lacZ/+*          N = 9; R8 = 1671

*w*; *Df(2R)BSC262/*+; *Rh6-lacZ/*+      N = 7; R8 = 961

*w*; *Df(2R)Exel6283/+*; *Rh6-lacZ/*+    N = 7; R8 = 1064

*w*; *esn^f0044^/Df(2R)BSC262*; *Rh6-lacZ/*+ N = 8; R8 = 1478

*w*; *esn^KO6^/Df(2R)BSC262*; *Rh6-lacZ/*+  N = 9; R8 = 1621

*w*; *esn^KO6^/Df(2R)Exel6283*; *Rh6-lacZ/*+   N = 9; R8 = 1225

-2D: graph bars from left to right; N = brains, R8 = total R8s counted in N brains

*w*; +; *GMR-gal4 UASDcr2 Rh6-lacz*/+           N = 8; R8 = 1495

*w*; *UAS-esnRNAi/+*; *Rh6-lacZ/*+               N = 6; R8 = 896

*w*; *UAS-esnRNAi/*+ ; *GMR-gal4 UAS-Dcr2 Rh6-LacZ/*+  N = 6; R8 = 945

-2E: *w*; *esn^KO6^/Df(2R)BSC262*; *GMR-gal4 UAS-Dcr2 Rh6-LacZ/*+

-2F: *w*; *esn^KO6^/Df(2R)BSC262*; *GMR-gal4 UAS-Dcr2 Rh6-LacZ/UAS-esn*

-2G: *w*; *esn^KO6^/Df(2R)BSC262*; *GMR-gal4 UAS-Dcr2 Rh6-LacZ/UAS-esnΔLIM*

-2H: graph bars from left to right; N = brains, R8 = total R8s counted in N brains

*w*; +; *GMR-gal4 UASDcr2 Rh6-lacz/*+                 N = 8; R8 = 1495

*w*; +; *UAS-esn/Rh6-lacZ*                         N = 10; R8 = 1730

*w*; +; *UAS-esnΔLIM /Rh6-lacZ*                    N = 10; R8 = 1046

*w*; *esn^KO6^/Df(2R)BSC262*;*GMR-gal4 UASDcr2 Rh6-lacz* /+        N = 9; R8 = 1177

*w*; *esn^KO6^/Df(2R)BSC262*; *GMR-gal4 UASDcr2 Rh6-lacz /UAS-esn*      N = 10; R8 = 1643

*w*; *esn^KO6^/Df(2R)BSC262*; *GMR-gal4 UASDcr2 Rh6-lacz /UAS-esnΔLIM*  N = 9; R8 = 1394

-2I: graph bars from left to right; N = brains, R8 = total R8s counted in N brains

*w*; +; *sens-gal4 UAS-UtrnCHGFP/*+               N = 8; R8 = 1434

*w*; +; *UAS-esn/Rh6-lacZ*                      N = 10; R8 = 1730

*w*; *esn^KO6^/Df(2R)BSC262*;*Rh6-lacZ/*+               N = 9; R8 = 1621

*w*; *esn^KO6^/Df(2R)BSC262*; *sens-gal4 UAS-UtrnCHGFP/UAS-esn*    N = 9; R8 = 1683

Figure 3

-3A: *w*; +; *GMR-gal4 UASDcr2 Rh6-lacz/*+

-3B: *w*; +; *GMR-gal4 UASDcr2 Rh6-lacz/UAS-fmiRNAi*

-3C: graph bars from left to right; N = brains, R8 = total R8s counted in N brains

*w*; *esn^KO6^/Df(2R)BSC262*; *Rh6-lacZ/*+        N= 7; R8 = 1223

*w*; +; *GMR-gal4 UASDcr2 Rh6-lacz/*+         N = 8; R8 = 1495

*w*; +; *GMR-gal4 UASDcr2 Rh6-lacz/UAS-fmiRNAi*    N = 8; R8 = 836

-3D: graph bars from left to right; N = brains, R8 = total R8s counted in N brains

*w*; *UAS-esnRNAi/*+ ; *GMR-gal4 UAS-Dcr2 Rh6-LacZ/*+         N = 6; R8 = 945

*w*; *FRT42D fmi^E59^/+*; *Rh6-lacZ*                     N = 6; R8 = 801

*w*; *UAS-esnRNAi/FRT42D fmi^E59^*; *GMR-gal4 UAS-Dcr2 Rh6-LacZ/*+    N = 7; R8 = 1052

-3E: graph bars from left to right; N = brains, R8 = total R8s counted in N brains

*w*; +; *GMR-gal4 Rh6-lacz/*+           N = 8; R8 = 1495

*w*; +; *UAS-esn/Rh6-lacZ*             N = 10; R8 = 1730

*w*; +; *GMR-gal4 Rh6-lacz/UAS-esn*       N = 10; R8 = 1558

-3F: *w*; +; *GMR-gal4 Rh6-lacz/UAS-esn*
