# Supplementary material for: The Cytoplasmic LIM Domain Protein Espinas Contributes to Photoreceptor Layer Selection in the Visual System
Source: Biology (Basel). 2020 Dec 14;9(12):466. doi: 10.3390/biology9120466 (PMC7764898; doi:10.3390/biology9120466)
Supplement: Supplementary file 1 [file biology-09-00466-s001.pdf]

SUPPLEMENTARY MATERIAL

Supplementary Material and Methods.

Genetics

Stocks used were: *w; GMR-myr-tdtom, F2BshortΔE1-GFP;* + (R8 marker [11]); *pk* $^{pk\text{-}sple13}$ (BDSC41790); *pk* ^{pk1} (BDSC367); *pk* $^{sple\text{-}1}$ (BDSC422). All genotypes analyzed in the Supplementary Figures are specified in detail in the Supplementary Appendix 1.

Photoreceptor phenotype quantification and statistics

Quantification and statisctical analysis of the overshoot phenotype was carried out as specified in the Materials and Methods of the manuscript.

Supplementary Appendix

Genotypes and quantification data of Supplementary Figures.

Figure S1

-1A, A′, B, B′, C, C′: *w; +; +*

-1D, D′: *w; esnMI03075-GFSTF.2/GMR-myr-tdtom, F2BshortΔE1-GFP; +*

-1E, E′: *w; esnMI03075-GFSTF.2/+; +*

Figure S2

- 2A: *w; FRT42D pk* $^{pk\text{-}sple\text{-}13}$*/+; Rh6-lacZ/+*
- 2B, D: *w; FRT42D pk* $^{pk\text{-}sple\text{-}13}$*/ FRT42D pk* $^{pk\text{-}sple\text{-}13}$*; Rh6-lacZ/+*
- 2C: graph bars from left to right; N=brains, R8=total R8s counted in N brains

w; +; Rh6-lacZ/+	N=6; R8=1435
w; FRT42D pk $^{pk\text{-}sple\text{-}13}$*/+; Rh6-lacZ/+*	N=9; R8=1760
w; FRT42D pk $^{pk\text{-}sple\text{-}13}$*/ FRT42D pk* $^{pk\text{-}sple\text{-}13}$*; Rh6-lacZ/+*	N=7; R8=1467

- 2E: *w; pk* $^{pk\text{-}1}$*/+; Rh6-lacZ/+*
- 2F, H: *w; pk* $^{pk\text{-}1}$*/ pk* $^{pk\text{-}1}$*; Rh6-lacZ/+*
- 2G: graph bars from left to right; N=brains, R8=total R8s counted in N brains

w; +; Rh6-lacZ/+	N=6; R8=1435
w; pk $^{pk\text{-}1}$*/+; Rh6-lacZ/+*	N=6; R8=1405
w; pk $^{pk\text{-}1}$*/ pk* $^{pk\text{-}1}$*; Rh6-lacZ/+*	N=7; R8=1594

- 2I: *w; pk* $^{sple\text{-}1}$*/+; Rh6-lacZ/+*
- 2J, L: *w; pk* $^{sple\text{-}1}$*/ pk* $^{sple\text{-}1}$*; Rh6-lacZ/+*
- 2K: graph bars from left to right; N=brains, R8=total R8s counted in N brains

w; +; Rh6-lacZ/+	N=6; R8=1435
w; pk $^{sple\text{-}1}$*/+; Rh6-lacZ/+*	N=7; R8=1263
w; pk $^{sple\text{-}1}$*/ pk* $^{sple\text{-}1}$*; Rh6-lacZ/+*	N=8; R8=1399

Supplementary Figures

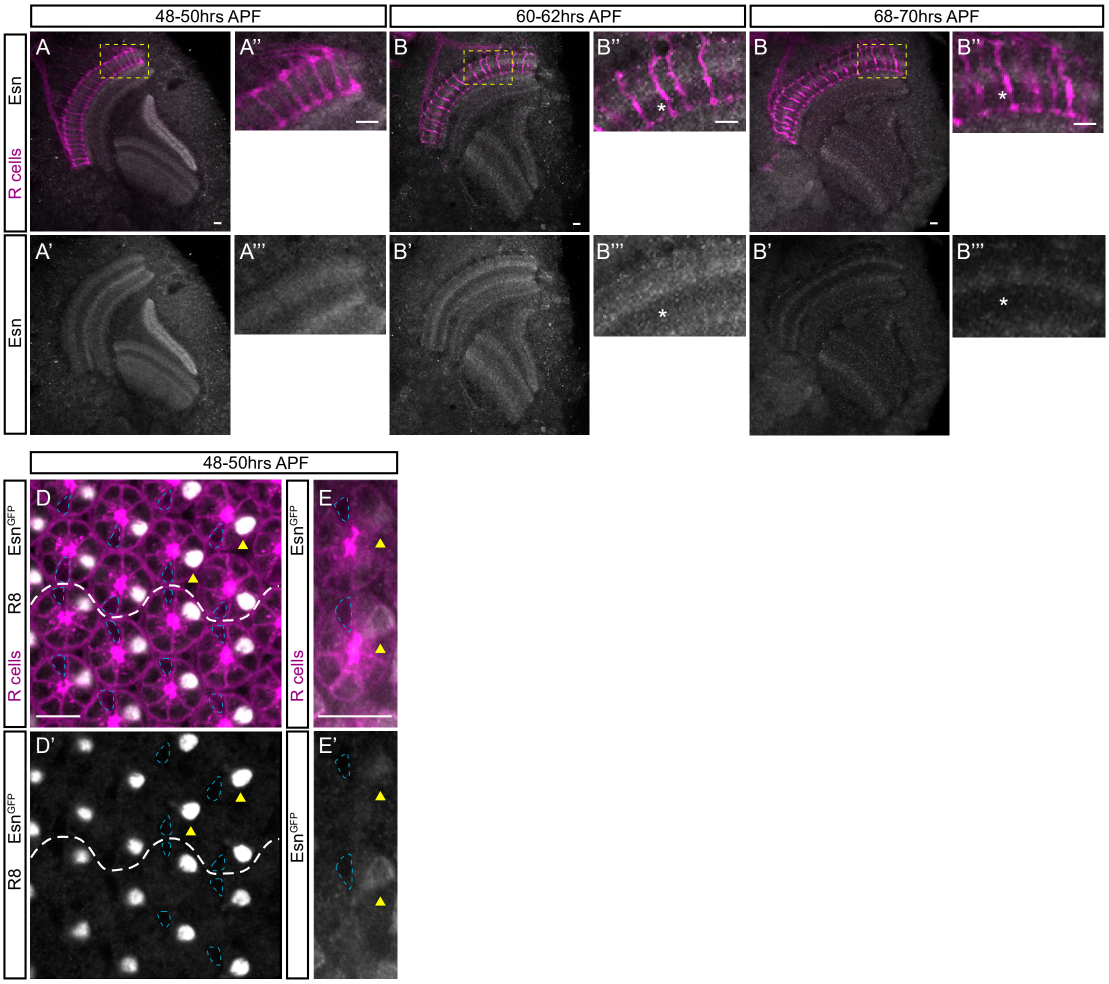

Figure S1. Extended Espinas (Esn) expression analysis. A-C′) Confocal sections of optic lobes at 48-50 (A-A′′′), 60-62 (B-B′′′) and 68-70 (C-C′′′) hrs APF stained with antibody against Esn (gray). Photoreceptor axons are labeled with antibody against Chaoptin (magenta). Yellow boxes indicate regions magnified in A′′, A′′′, B′′, B′′′, C′′, C′′′. During this period the R8 sends a filopodia to the final layer (48-50hrs APF) and transforms into a synaptic terminal (60-62 to 68-70hrs APF). Note Esn signal between the R8 and the R7 terminals in all stages. Absence of enrichment of signal along the path of the R8 filopodia (A-A′′′), the R8 axon or terminal (B-C′′′), makes it difficult to discern Esn expression in the R8 from Esn expression in other medulla neurons. A-A′′′) At 48-50hrs APF the R8 growth cone sitting in the temporary layer sends a thin filopodia to the final layer. Even in the absence of Esn expression in other neurites, it might be hard to detect Esn in the filopodia, such a thin and dynamic structure, in fixed tissue. Indeed, the filopodial step has been described through live imaging. B-B′′′) At 60-62hrs APF, the R8 terminal starts being discerned as an enlargement (asterisk in B′′). There is Esn signal right above this structure but no enrichment on the R8 axon. C-C′′′) At 68-70hrs APF, the R8 synaptic terminal (asterisk in C′′) is mostly formed. The strongest Esn signal lies above this structure, and actually, the Esn signal seems to be stronger between photoreceptor axons than on the path of the R8/R7 axon. D-E) Confocal images of 48-50hrs APF retinas. D-D′) Projection of three several confocal sections showing the outline of photoreceptor cell bodies (magenta, labelled with the transgene *GMR-myr-tdtom*), the R8 nuclei and Esn protein (gray, both visualized with antibody against GFP, detecting the R8 nuclei through the transgene *F2Bshort-ΔE1-GFP* and Esn protein through the Esn-GFP protein trap *esnMI03075-GFSTF.2).* In this image we characterize the position of the R7 and R8 cell bodies in the photoreceptor cluster of each ommatidia. R7 cell bodies (outlined with a cyan dashed line) face each other across the equator (white dashed line). The R8 nuclei (yellow arrowheads) are the second cell to the right of the R7. In D′ the GFP channel is shown in gray. Only the R7s in the right side of the panel are outlined to allow comparison to the left side of the panel, where it is obvious that the absence of Esn signal identifies the R7 cell bodies. The strong signal in the R8 nuclei masked the expression of Esn. E-E′) Confocal

section of retina showing the outline of photoreceptor cell bodies (magenta, using antibody against Chaoptin) and Esn (gray, detected with antibody against GFP, through the Esn-GFP protein trap *esnMI03075-GFSTF.2).* In this experiment the R8 nuclei is not labelled, which permits the assessment of the Esn signal. At this stage the R8 cell (yellow arrowhead) shows the highest levels of Esn protein in the cluster. Scale bars 10 microns.

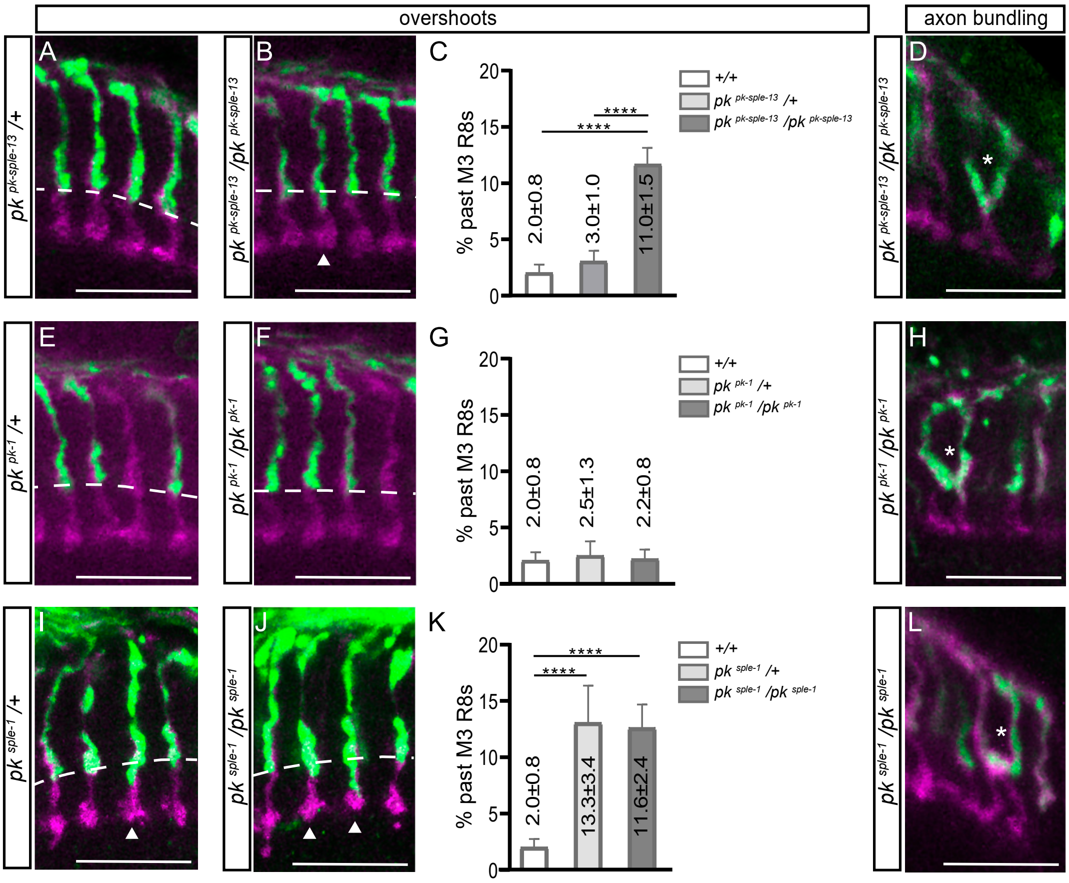

Figure S2. Analysis of *prickle (pk)* mutant alleles. *pk* has two isoforms, *pk* and *sple,* with *pk* being the most similar isoform to *esn*. The alleles under study are: $pk^{pk\text{-}sple\text{-}13}$ (A-D) (also known as pk^{sple13}) a null allele that eliminates both isoforms; pk^{pk1} (E-H) (also known as pk^{1}) which eliminates the *pk* isoform; and $pk^{sple\text{-}1}$ (I-L) which eliminates the *sple* isoform. The balance between *pk* and *sple* isoforms is essential in the differentiation of the R3 and R4 photoreceptors in the context of planar cell polarity [1, 2]. Thus, we think that in addition to *pk* specific PCP functions (mediated by *pk* and *sple* isoform balance), it might have partially redundant function with *esn*. Confocal images show the photoreceptor array with R7 and R8 axons in magenta (antibody against Chaoptin) and R8 also labelled in green (Rh6-lacZ transgene). Graphs show quantification of phenotypes and the statistical significance of relevant comparisons. Analysis of the R8 axons revealed two phenotypes: overshoots past M3 (A-C, E-G, I-J) and axon bundling (D, H, L). Overshoots past the M3 layer were observed when eliminating the *sple* isoform even in heterozygous condition (I-K) but not the *pk* isoform (E-G). This suggests that the *sple* isoform plays a role in role R8 layer selection. Interestingly, the percentage of R8 overshoots is very similar to that of *esn* mutants. Given that *pk* isoform is the closest to *esn*, we interpret the absence of overshoots in the pk^{pk1} animals as *esn* being able to compensate for the absence of the *pk* isoform, but not of the *sple* isoform. Instances of axon bundling were common to all three alleles in homozygosis (asterisk in D, H, L). This phenotype has also been reported in transheteroallelic hypomorphic fmi animals (fmi[E86]/<fmi<[1]) and enhanced by removing one copy of Gogo [3]. The phenotypes observed suggest that the *pk* gene plays a role in R8 wiring and that it could be related to signaling downstream of *fmi*. The fact that *esn* and *pk* are genes duplicated in tandem precluded us from doing additional experiments such as double mutant analysis. **** p<0.0001. Scale bars 10 microns.

1. Collu, G. M.; Jenny, A.; Gaengel, K.; Mirkovic, I.; Chim M-L.; Weber, U.; Smith M. J.; Mlodzik, M. Prickle is phosphorylated by Nemo and targeted for degradation to maintain Prickle/Spiny-legs isoform balance

during planar cell polarity establishment. *PLoS Genet*. **2018**, *14*, 1–25. https://doi.org/10.1371/journal.pgen.1007391

2. Lin, Y. Y.; Gubb, D. Molecular dissection of Drosophila Prickle isoforms distinguishes their essential and overlapping roles in planar cell polarity. *Dev. Biol.* **2009**, *325*, 386–399. https://doi.org/10.1016/j.ydbio.2008.10.042
3. Hakeda-Suzuki, S.; Berger-Müller, S.; Tomasi, T.; Usui, T.; Horiuchi, S.; Uemura, T.; Suzuki, T. Golden Goal collaborates with Flamingo in conferring synaptic-layer specificity in the visual system. *Nat. Neurosci.* **2011**, *14(3),* 314–323. https://doi.org/10.1038/nn.2756
